# Centrin diversity and basal body patterning across evolution: new insights from *Paramecium*

**DOI:** 10.1242/bio.024273

**Published:** 2017-04-21

**Authors:** Anne Aubusson-Fleury, Guillaume Balavoine, Michel Lemullois, Khaled Bouhouche, Janine Beisson, France Koll

**Affiliations:** 1 Institute for Integrative Biology of the Cell (I2BC), Cell Biology Department, CEA, CNRS, Université Paris Sud, Université Paris-Saclay, 1 Avenue de la Terrasse, Gif sur Yvette 91198, France; 2 Institut Jacques Monod, Evolution and development of Metazoa, UMR 7592, CNRS/Université Paris Diderot, 15 rue Hélène Brion, Paris 75013, France; 3 INRA, UMR 1061 Unité de Génétique Moléculaire Animale, Université de Limoges, IFR 145, Faculté des Sciences et Techniques, Limoges 87060, France

**Keywords:** Ciliary centrins, Basal body assembly, Basal body anchoring, Ciliated epithelia polarity, Centrin evolution

## Abstract

First discovered in unicellular eukaryotes, centrins play crucial roles in basal body duplication and anchoring mechanisms. While the evolutionary status of the founding members of the family, Centrin2/Vfl2 and Centrin3/cdc31 has long been investigated, the evolutionary origin of other members of the family has received less attention. Using a phylogeny of ciliate centrins, we identify two other centrin families, the ciliary centrins and the centrins present in the contractile filaments (ICL centrins). In this paper, we carry on the functional analysis of still not well-known centrins, the ICL1e subfamily identified in *Paramecium*, and show their requirement for correct basal body anchoring through interactions with Centrin2 and Centrin3. Using *Paramecium* as well as a eukaryote-wide sampling of centrins from completely sequenced genomes, we revisited the evolutionary story of centrins. Their phylogeny shows that the centrins associated with the ciliate contractile filaments are widespread in eukaryotic lineages and could be as ancient as Centrin2 and Centrin3.

## Abstract (precis)

**Summary:** Functional and phylogenetic analyses reveal the existence of five centrin families and show that basal body patterning in *Paramecium* requires a third centrin present in many eukaryote lineages.

## INTRODUCTION

The role of centrins in centriole/basal body/spindle pole body duplication (Salisbury et al., 1984; Wright et al., 1985; Baum et al., 1986) is a long-standing debate. It is now broadly agreed that centrins may be dispensable for centriole duplication, but are required for basal body biogenesis. This primary association of centrins with basal bodies is consistent with the generally admitted hypotheses that a cilium and associated basal body or basal body-like structure were present in the common ancestor of eukaryotes, and that basal body function is ancestral, whereas the presence of the centrosome is specific to the Holozoa clade (Azimzadeh and Bornens, 2007; Hodges et al., 2010; Carvalho-Santos et al., 2011). As stated by Dantas et al. (2012), centrins “play a major role in ensuring the duplication and appropriate functioning of the ciliary basal bodies in ciliated cells”. This statement applies to multiciliated cells, whether in protists or in metazoan, and still to be fully understood is the precise function of centrins in the development and functional organisation of ciliatures.

Studies in flagellated/ciliated unicellular organisms such as *Chlamydomonas*, *Paramecium*, and *Tetrahymena*, have long established the key role of the conserved ubiquitous centrins, Centrin2/Vfl2 and Centrin3/cdc31 in basal body duplication (Wright et al*.*, 1985; Koblenz et al., 2003; Ruiz et al., 2005; Stemm-Wolf et al., 2005). While metazoa and plants possess a small number of centrins, essentially centriolar centrins Centrin2 and/or Centrin3, a diversity of centrins is present in unicellular organisms (Mahajan et al., 2008; Nagamune et al., 2008; Stemm-Wolf et al., 2005; Lauwaet et al., 2011).

By far the richest in centrin diversity is *Paramecium tetraurelia* (Gogendeau et al., 2008). Although this gene multiplicity is largely due to the three successive genome duplications (Aury et al., 2006), it nevertheless involves a wide functional diversity, with four centrins exclusively localised at basal bodies and involved in their assembly and positioning (Ruiz et al., 2005), one centrin involved in the control of ciliary voltage-gated (CVGC) Ca^++^-mediated channels (Gonda et al., 2004), and 36 grouped in a large family: the infraciliary lattice (ICL) centrins (Gogendeau et al., 2008). This ICL is a subcortical, contractile network (Garreau de Loubresse et al., 1988, 1991; Klotz et al., 1997), built up with SfI1/centrin filaments (Gogendeau et al., 2007), which subtends the whole cell surface and plays a role in Ca^++^ homeostasis (Sehring et al., 2009). The ICL centrins are divided into 10 phylogenetic subfamilies. Functional studies of one representative of each of the 10 subfamilies have shown that they localise all along the ICL and are essential for its biogenesis, but that most of them are dispensable for cell survival. In contrast, ICL1e, one of the seven members of the ICL1e subfamily, which localises not only to the ICL, but also in close association with the basal bodies, is essential for cell survival and basal body biogenesis (Gogendeau et al., 2008). In view of the fundamental role of this centrin, it seemed of interest to analyse further the role of all the members of ICL1e subfamily.

Contractile centrin networks are found in many ciliate species and constitute one of the major layers of their superficial cytoskeleton (Fleury et al., 1992). But are the ICL centrins specific for these contractile networks? What is their evolutionary origin? Starting from *Paramecium* and other ciliates, we asked for the origin of centrin diversity and function among eukaryotes.

## RESULTS

### Five functional centrin families can be identified in *Paramecium*

**Fig. 1.**
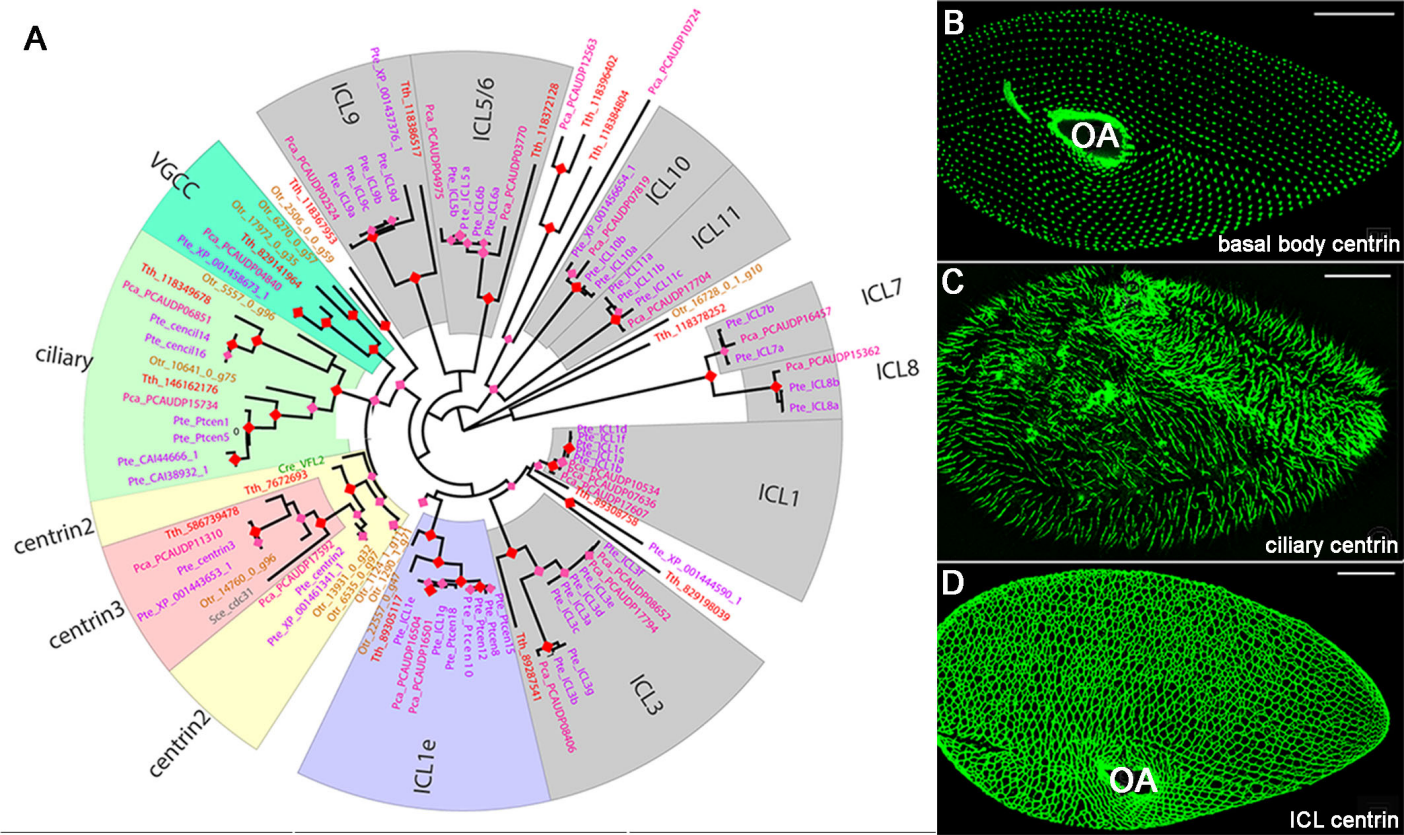
**Ciliate centrins: phylogeny and localisation in *Paramecium*.** (A) Maximum-likelihood unrooted tree of ciliate centrins. The robustness of the nodes is coded by coloured diamonds: pink for aLRT scores superior to 75%, red for aLRT scores superior to 95%. Boxes indicate well supported sub-groups, from either the phylogenetical or functional point of view. Pte (purple): *Paramecium tetraurelia*; Pca (pink): *Paramecium caudatum*; Tth (red): *Tetrahymena thermophila*; Otr (brown): *Oxytricha trifallax*. Two outgroup sequences for Centrin2 and Centrin3 families have been added: Cre/VFL2 (green), *Chlamydomonas reinhardti* and Sce/cdc31 (grey), *Saccharomyce cerevisiae*. (B-D) Confocal images. GFP-tagging of representatives of these diverse centrins reveals their three major localisations: at basal bodies (B), in cilia (C) and along the infraciliary lattice (D) labelled by GFP-Centrin2, GFP-Cent14 and GFP-ICL1e, respectively. OA, oral apparatus. Scale bar: 20 µm.

We revisited the phylogenetic relationships of centrin families in Ciliata in using the completely sequenced organisms: two *Paramecium* genomes, *P. tetraurelia* used in this study and *P. caudatum* which has undergone two less whole genome duplications resulting in less redundancy (McGrath et al., 2014); *Tetrahymena thermophila*, another species from the same taxonomic group (Adl et al., 2012); and an evolutionary more distant species, *Oxytricha trifallax*. 50 genes encoding centrin were found in *P. tetraurelia*, 22 in *P. caudatum*, 15 in *T. thermophila*, and 12 in *O. trifallax*. The resulting tree (Fig. 1A) shows a number of branches with sequences representative of the four species, likely representing ancestral ciliate families. Combining phylogenetic analysis and functional data, five main functional families can be defined (Fig. 1A). The first two families correspond to the classical basal body associated centrins, Centrin2 (Centrin2/VFL2) and Centrin3 (Centrin3/cdc31) (Ruiz et al., 2005). The phylogenetic position of four closely related paralogues (PtCen1, 5, 14 and 16) suggests that they could form a new family. PtCen1 and PtCen14 were identified in the ciliary proteome (Cildb, Arnaiz et al., 2014). GFP-tagged PtCen14 specifically localised to cilia (Fig. 1C) and its RNAi depletion resulted in altered swimming behaviour (Fig. S1). These centrins can thus be considered as ciliary centrins. The function of such a ciliary centrin has already been analysed in another protist, *Trypanosoma* (Wei et al., 2014). The fourth family contains the *P. caudatum* centrin required for the function of a ciliary voltage gated calcium channel (VGCC centrin, Gonda et al., 2004) and its *P. tetraurelia* ortholog, which is supposed to fulfill the same function. Finally, the ICL centrins, required for the assembly of the contractile network (Gogendeau et al., 2008) are grouped into a large functional fifth family (Fig. 1D). These sequences, quite divergent between ciliate species, do not form a common cluster. However, sequences representatives of *P. caudatum* and *Tetrahymena* are found in most branches corresponding to the previously defined ICL centrin subfamilies of *P. tetraurelia*, suggesting that they diverged before the emergence of these two genera.

Sequences representative of the four species are found in several well-supported branches: Centrin2, Centrin3, Ciliary and VGCC centrins. Strikingly, within the ICL centrins, only the ICL1e subfamily also comprises at least one sequence from all species including the more distantly related genus, *O. trifallax*. This suggests that ICL1e centrins could be ancient. It then seemed of interest to re-investigate the role of all members of this ICL1e subfamily in basal body biogenesis and organisation of the ICL.

### Localisation of the ICL1e centrins

The ICL1e subfamily comprises seven genes coding for centrins corresponding to three ohnolog pairs, i.e. gene duplicates originating from whole genome duplication (ICL1e and ICL1 g; Cen8 and Cen10; Cen12 and Cen15) and a single gene, Cen18. In order to ascertain their respective localisation and function, a representative from each ohnolog group (ICL1e, Cen10, Cen15, Cen18) was GFP-tagged.

**Fig. 2.**
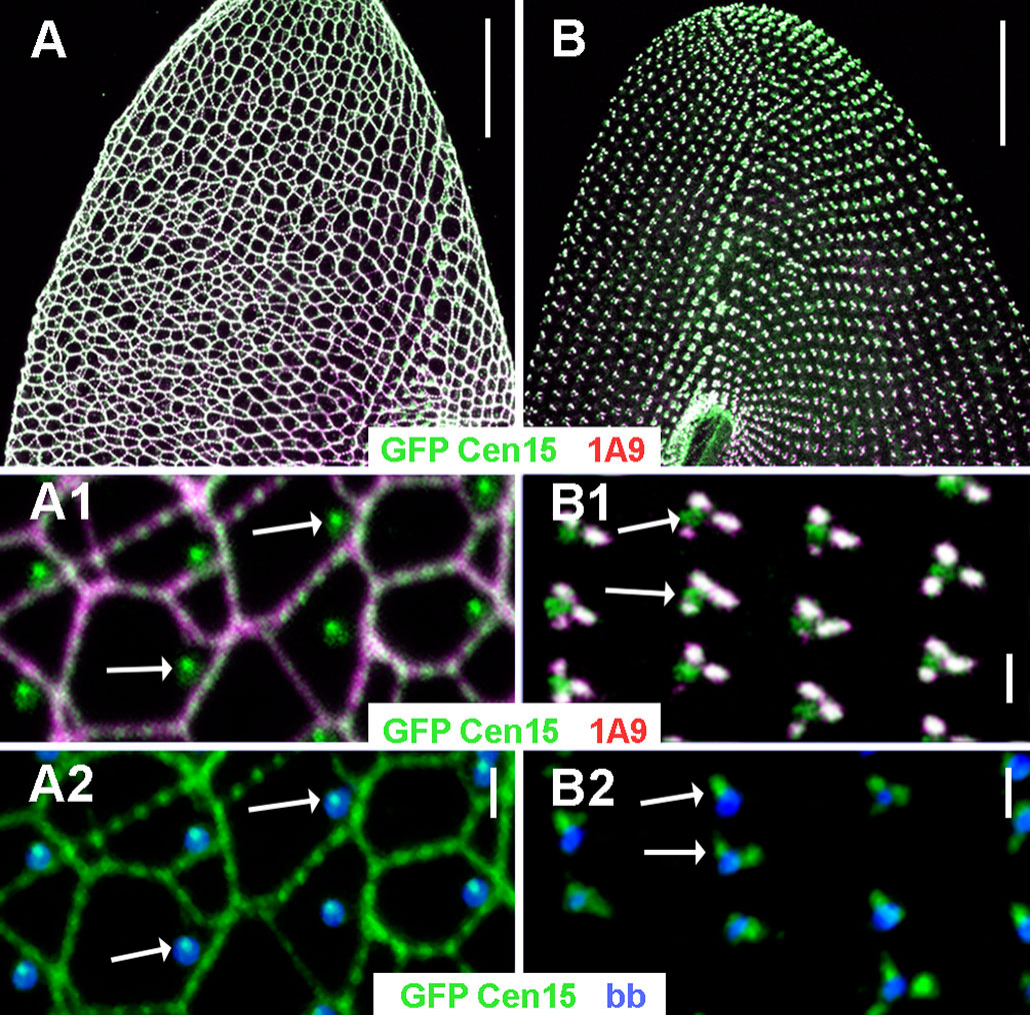
**Wild-type and ICL-less (BP) cells: respective localisations of ICL1e, ICL centrins and basal bodies.** Confocal images. (A) Wild-type cell triple-labelled by GFP-ICL1e (green), the anti-ICL 1A9 (magenta) and the anti-tubulin PolyE to label basal bodies (blue): ventral side showing the ICL network. Insets: the GFP-labelling shows a dotted pattern along the meshes of the network (A1) and a singular dot anterior right to each basal body (A2). (B) Two BP cells double-labelled with GFP-ICL1e (green) and 1A9 (B and B1) and with GFP-ICL1e and the PolyE (B2). (B1) remnants of the ICL network are labelled by both 1A9 and GFP-ICL1e, except for a dot which is not decorated by 1A9. (B2) The GFP-ICL1e dot is detected close to each basal body as in the wild type. Arrows: ICL1e dot. Scale bar: 20 µm in A, B; 1 µm in A1-2, B1-2.

GFP-fusion constructions were introduced into two types of cells, the wild type (WT) and a mutant (BP) deleted for the Sfi-like centrin-binding protein and devoid of ICL. BP cells retain only a remnant of the infraciliary lattice closely associated to each basal body or basal body couplet, called ICL-organising centre (ICLOC) as it nucleates the assembly of the ICL under rescue conditions (Beisson et al., 2001). ICL centrins are labelled by the monoclonal 1A9 which recognises the most acidic of the ICL centrins (Beisson et al., 2001). In WT cells (Fig. 2A), both GFP-ICL1e centrins and 1A9 label the whole network, but the two labels do not exactly overlap: the GFP-ICL1e labelling appears as discrete dots localised along a linear structure decorated by 1A9. In addition, all GFP-tagged members of the ICL1e subfamily localise as a dot near each single or paired basal body not recognised by the monoclonal 1A9. These results demonstrate that the 1A9 antibody does not recognise the ICL1e centrins. In BP cells, the ICLOC stands out as a bipartite structure, with a GFP-ICL1e centrin-labelled dot close to the basal body and not recognised by the monoclonal 1A9, and a small cluster of patches of less-defined shape, labelled by both 1A9 and the GFP-ICL1e centrins (Fig. 2B). The ICL1e dot, present in both WT and BP cells, thus appears as a constitutive basal body-associated structure. Because all the seven ICL1e centrins colocalise at this dot, we will call it the ICL1e complex.

To analyse the role of this ICL1e complex, we wanted to (1) ascertain the relative timing of duplication of basal body and ICL1e complex, (2) analyse the effect of ICL1e centrins depletion on basal body and ICL biogenesis and (3) investigate the possible functional relationships between the ICL1e complex and the basal body centrins, Centrin2 and Centrin3.

### Timing of duplication of the ICL1e complex, the basal bodies and the ICL

All over the cell surface, basal bodies, organised as antero-posterior rows, are inserted in a submembranar cytoskeleton, the epiplasm, segmented in longitudinal files of ‘cortical units’, within which one or two basal bodies are anchored (Iftode et al., 1989; Aubusson-Fleury et al., 2013). During division, the assembly of new basal bodies generates an alternance of parental and new basal bodies along each row. At the cell level, this duplication follows a precise spatio-temporal pattern which exactly restores the distribution of basal bodies in the two daughter cells (Fig. S2-1).

**Fig. 3.**
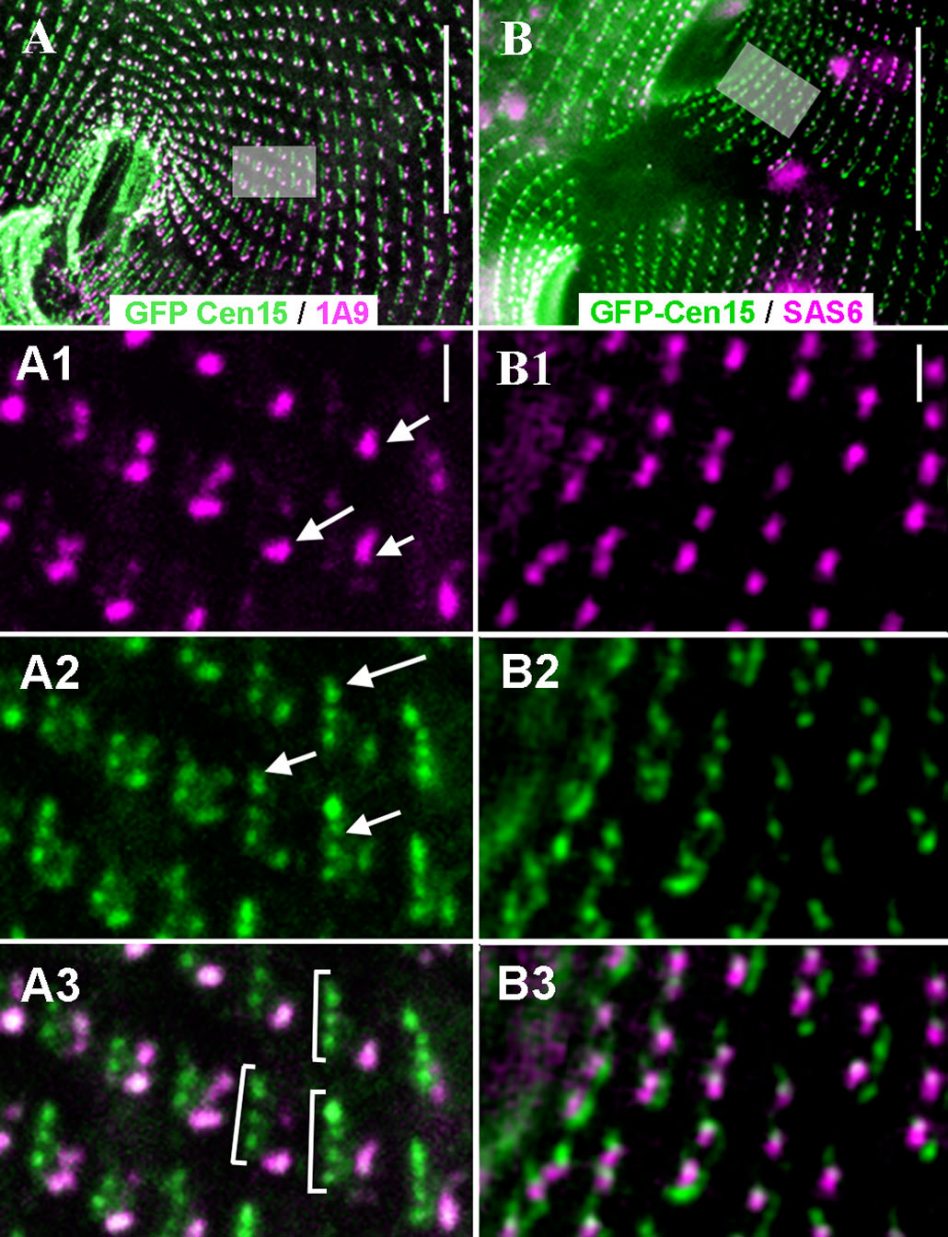
**The concerted duplication of basal bodies and ICL1e complexes in ICL-less BP cells.** Epifluorescence images. (A-A3) Sequential assembly of the ICL1e complex and other ICL components. At the beginning of division, new basal bodies are assembled anterior to parental ones, resulting in a series of closely juxtaposed basal bodies (see Fig. S2A). In this dividing BP cell (A) expressing GFP-Cen15 (green) and labelled by 1A9 (magenta), basal body duplication has already begun resulting in series (brackets in A3) of new ICL1e complexes (arrows in A2) associated with just duplicated basal bodies. By contrast, the 1A9 labelling (arrows in A1) is only associated with the parental (posterior) basal bodies of each series. (B-B3) Respective timings of duplication of basal bodies and ICL1e dots. In this cell co-expressing GFP-Cen15 (green) and RFP-SAS6 (magenta), on both sides of the fission furrow, an ICL1e complex flanks anteriorly each SAS-6 dot (magenta) marking all basal bodies, whether pre-existing or just formed. Scale bars: 20 µm in A, B; 1 µm in A1-3, B1-3.

In dividing BP cells, the GFP-ICL1e complex duplicates first, while the 1A9-reactive spots will re-assemble later (Fig. 3A). This can also be observed in WT cells at the onset of division (Fig. S2-2). This stepwise assembly shows that the duplication of the ICLOC over cell divisions in BP cells, as well as of the ICL in WT cells, follows the duplication of the ICL1e complex.

We then compared the time of appearance of new ICL1e dots to that of SAS-6, the earliest known marker of nascent basal bodies in *Paramecium* (Jerka-Dziadosz et al., 2012). In cells expressing both RFP-SAS-6 and GFP-ICL1e, the SAS-6 and ICL1e foci co-localise at all new single basal bodies (Fig. 3B): the ICL1e complex that accompanies the new basal bodies as early as SAS-6 is detectable.

Therefore, the ICL1e complex duplicates along with the basal bodies before the assembly of the other components, to reconstitute the interphasic ICL network in WT cells or the ICLOC in BP cells.

### Effects of ICL1e depletion on the cortical organisation of the basal bodies

In order to analyse more precisely the role of the ICL1e complex, we depleted WT and BP cells by RNAi of representatives of each ohnolog group. Possible off-target effects were systematically screened for each construction (Fig. S3-2).

**Fig. 4.**
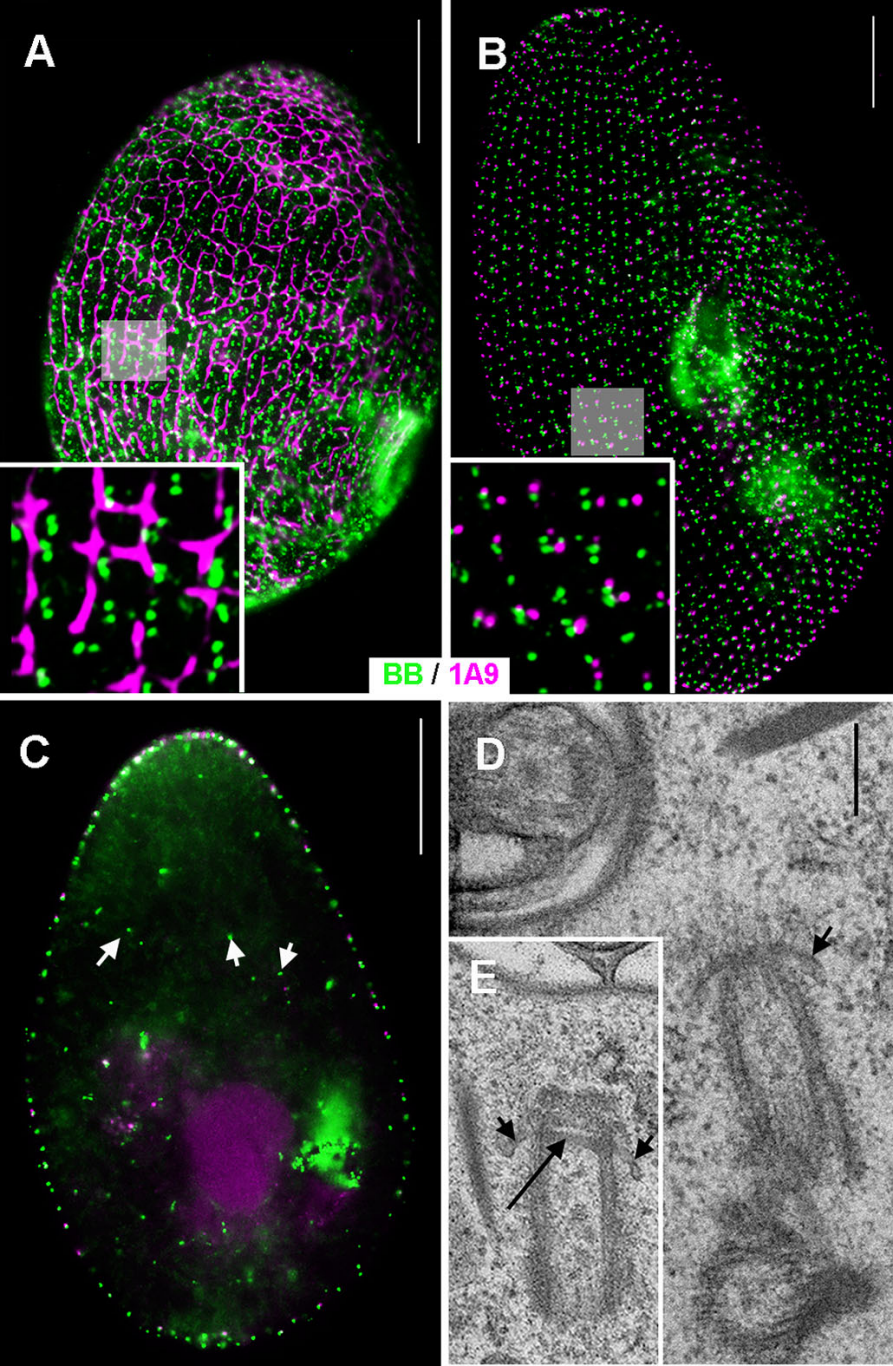
**Effects of ICL1e inactivation on the basal body pattern.** Epifluorescence images. (A) In wild-type cells, by the first/second division upon inactivation, the ICL network (magenta) is disrupted and mis-positioned basal bodies (1D5, green) are detected. Scale bar: 20 µm; inset: ×3.5. (B) In BP cells, by the first /second division upon inactivation, basal bodies without associated ICL remnants (magenta) are detected (1D5 green, arrows). Scale bar: 20 µm; inset: ×3. Insets in A and B are outlined by grey squares in corresponding figures. (C) Numerous internal basal bodies are detected (arrows). Scale bar: 20 µm. (D,E) Transmission electron microscopy of ICL1e-inactivated wild-type cell. The unanchored basal bodies appear fully developed with a transition zone (large arrow) and associated ring structure (short arrows). Scale bar: 200 nm.

RNAi knockdown of ICL1e centrins in WT cells first causes a stripping of the ICL network (Fig. 4A), then scattered disorders in the organisation of basal body rows, and cell growth arrest after a few divisions. In BP cells, the earliest effect, soon after the first division under RNAi conditions, is the absence of ICLOC on newly assembled basal bodies (Fig. 4B) then followed by disorganisation of basal body rows after two or three divisions. In both BP and WT cells, patches of supernumerary mislocalised basal bodies appear. These erratic basal bodies are not anchored to the cell surface and may be found intra-cytoplasmically (Fig. 4C). At the ultrastructural level, the unattached basal bodies present a transition zone (Fig. 4D,E). Depletion of ICL1e thus does not impair duplication per se (since new basal bodies appear fully developed), but affects the insertion of newly formed basal bodies at the cell surface, a syndrome similar to the effect of inactivation of Centrin3 (Ruiz et al., 2005; Aubusson-Fleury et al., 2012).

The silencing of each member of the ICL1e subfamily yielded the same defective phenotype as induced by ICL1e centrin inactivation (Gogendeau et al., 2008). All the members of the ICL1e subfamily are essential to trigger the ICLOC/ICL assembly and the positioning of the new basal bodies. They do not complement each other and thus form a single functional complex.

### Topological relationships between ICL1e and the basal body Centrin2 and Centrin3, during division

ICL1e, like Centrin2 and Centrin3 (Ruiz et al., 2005), are required for proper basal body positioning/anchoring in *Paramecium*. To ascertain the chronology of appearance of the three types of centrins, we compared it to that of the detection of basal body labelling at three successive stages of duplication of the cortical units (Fig. S2-1): at the onset of duplication just before detection of the new basal bodies within the cortical units, after duplication in newly individualized units with single basal bodies, and at the end of duplication after the formation of units with single and/or paired basal bodies.

In interphase cells, Centrin2 localises within the basal body cylinder, while Centrin3 localises anterior to single basal bodies, and between the two basal bodies when paired (Ruiz et al., 2005). At that stage, the localisations of ICL1e and Centrin3 are similar.

**Fig. 5.**
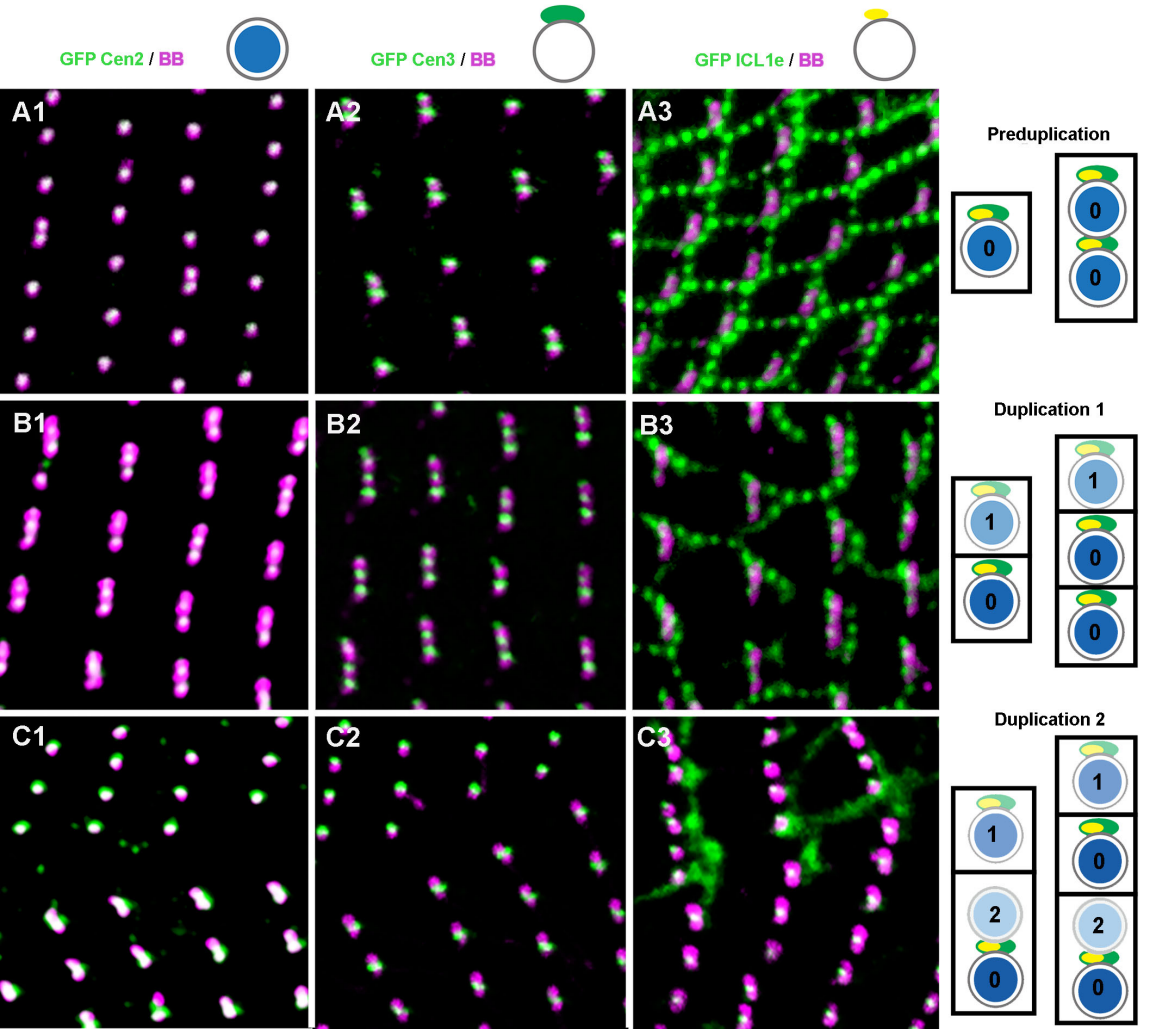
**Topological relationships between Centrin2, Centrin3 and ICL1e during basal body duplication.** Confocal images. Three successive stages of basal body duplication are illustrated in cells expressing GFP-Centrin2 (A1-C1), GFP-Centrin3 (A2-C2) or GFP-ICL1e (A3-C3) and labelled with the ID5 antibody. (A) Pre-duplication, (B) duplication 1 leading to one basal body per cortical units, and (C) duplication 2, resulting in paired basal bodies in some units. The scheme to the right summarises the specific behaviour of each of the three centrins at successive stages. Circles: basal bodies; colour intensities represent successive basal bodies generations (0, parental basal bodies; 1, basal bodies from duplication 1; 2, basal bodies from duplication 2); blue, Centrin2; green, Centrin3; yellow, ICL1e. While the Centrin2 labelling (A and blue) is superimposed to the basal bodies at all three stages, Centrin3 (2 and green) and ICL1e (3 and yellow) all localise anterior of all basal bodies during the pre-duplication stage (A), and anterior of the single and of the sole posterior basal body in paired basal bodies at other stages (B and C). Each image represents a 10×10 µm area.

At the onset of unit duplication, before basal body duplication, a new spot of Centrin3 appears anterior to the anterior basal body of pairs (Fig. 5). Thus, at this stage, an anterior Centrin3 spot is associated to all paired and single basal bodies. Interestingly, the ICL1e labelling exactly follows the same pattern as Centrin3 (Fig. 5A).

A new spot of Centrin2 then appears just anterior to parental basal bodies (not shown). The new basal body is detected soon after by labelling with the ID5 antibody. The successive detection of a GFP-Centrin2 and of an ID5 spot suggests that Centrin2 is an early marker of basal body assembly, as already demonstrated for the centriole (Azimzadeh et al., 2009). As soon as the new basal body is detected, it is associated with an anterior spot of Centrin3 and ICL1e (Fig. 5B).

At the end of cell division, in some units, basal bodies duplicate to yield pairs: a GFP-Centrin2 spot first appears, then followed by the detection of a new basal body. Neither new GFP-Centrin3 nor GFP-ICL1e spots are detected in association with this new anterior basal body (Fig. 5C).

These observations show that, while Centrin2, localised inside the basal body, is an early marker of basal body assembly, the recruitment of Centrin3, localised outside the basal body (Ruiz et al., 2005) and of ICL1e at a new anterior paired basal body may be delayed until the next duplication: the full assembly and anchoring of a basal body do not need its association with Centrin3 and the ICL1e complex.

### Interactions between ICL1e and Centrin2 and Centrin3

**Fig. 6.**
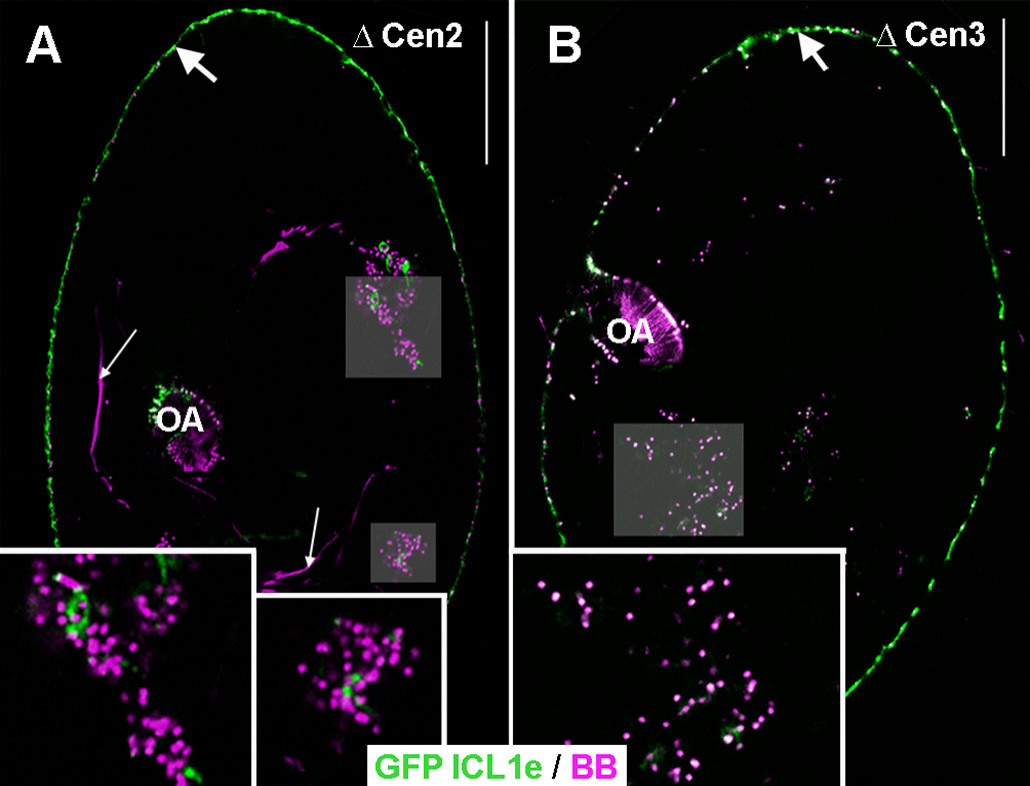
**Localisation of GFP-ICL1e upon inactivation of Centrin2 and Centrin3.** Confocal images. Green, GFP-ICL1e; magenta, basal bodies labelled with ID5. Insets are indicated by grey squares. (A) Upon inactivation of Centrin2, intra-cytoplasmic basal bodies are devoid of the ICL1e labelling. (B) In contrast, upon inactivation of Centrin3, intra-cytoplasmic basal bodies display the ICL1e labelling. In both cases, ICL remnants are detected at the cell surface (large arrows). Thin arrows, intracytoplasmic microtubules. OA, oral apparatus. Scale bar: 20 µm; ×2 in insets.

**Fig. 7.**
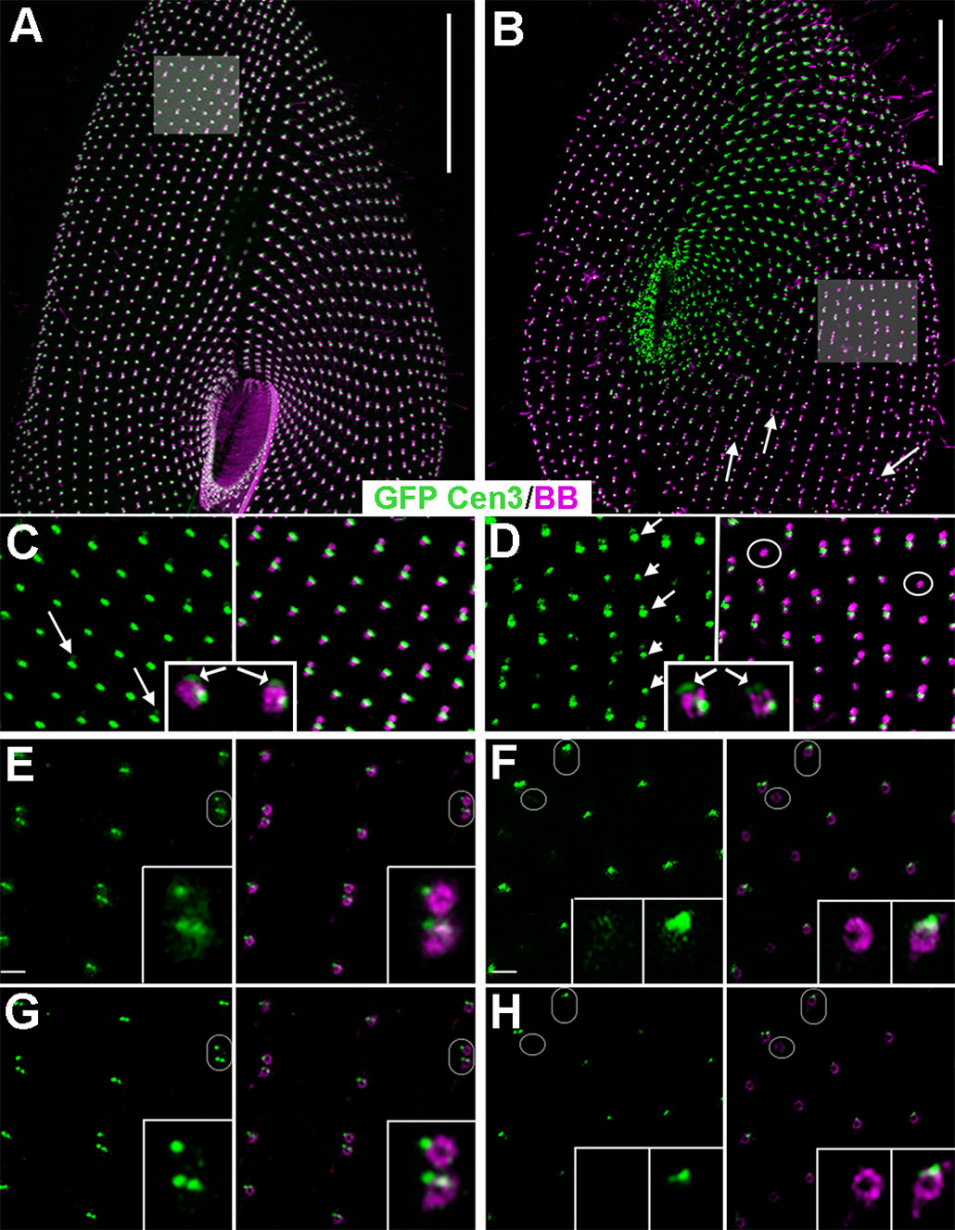
**Localisation of GFP-Centrin3 upon inactivation of ICL1e.** (A-D) Confocal and (E-H) STED images. Cells expressing GFP-Centrin3 (green) were immunolabelled with ID5 (magenta) to decorate the basal bodies. (A,C,E,G) Control, (B,D,F,H) ICL1e-depleted cell. Scale bars: 20 µm. The areas shown in C and D are outlined in A and B. (A) Wild-type cell, Centrin3 is associated with all basal bodies. (C) All spots of Centrin3 associated with single or paired basal bodies display a similar intensity. By the anterior basal body of the pairs, an additional faint spot is detected (arrows in C) suggesting a pre-duplication stage. Inset in C, lateral view of a basal body by STED imaging; the base and tip localisations (arrow) of the Centrin3 are detected. (E) Non-deconvoluted and (G) deconvoluted STED images of another Centrin3-expressing cell. The part magnified in the insets is outlined with an ellipse. Two Centrin3 spots anterior to single basal bodies and posterior basal body of paired ones, as well as an additional anterior spot associated with the anterior basal body of each pair are detected. Inset in E, the three spots are surrounded by a faint cloud of GFP-material, which could correspond to the GFP-Centrin3 at the tip of the basal body. After deconvolution (G), the three spots are clearly identified, but the surrounding cloud is no longer detected. Scale bars: 0.5 µm. (B) ICL1e-depleted cell. Basal bodies are less regularly aligned, and extra basal bodies are observed (arrows). (D) An alternance of strong (long arrows) and weak (short arrows) fluorescence of the GFP-Centrin3 spots associated with the basal bodies is observed along the antero-posterior rows. Some basal bodies have almost lost their Centrin3 (circles). Inset in D, lateral view of a basal body by STED imaging: the double (at the base and the tip) localisation (arrow) of the Centrin3 is detected but with a variable intensity depending upon the basal body. (F) Non-deconvoluted and (H) deconvoluted STED images of another cell. Left insets are outlined by a circle and right insets by an ellipse. After ICL1e depletion, the number and size of the GFP-Centrin3 spots are smaller. The right anterior Centrin3 spot is lost in most basal bodies, and some basal bodies have no spot at all while a tiny cloud of GFP-Centrin3 is still detected (F, left inset).

RNAi knockdown of either Centrin2 or Centrin3 disturbs basal body attachment to the cell surface, yielding intra-cytoplasmic basal bodies (Ruiz et al., 2005). Double-labelling of ICL1e and basal bodies shows that, upon inactivation of Centrin2, no GFP-ICL1e is found in association with intra-cytoplasmic basal bodies (Fig. 6A). Thus, although the two markers do not colocalise, Centrin2 is required for ICL1e localisation. In contrast, upon inactivation of Centrin3, GFP-ICL1e still localises at intra-cytoplasmic basal bodies (Fig. 6B). In the reverse situation, i.e. inactivation of ICL1e on GFP-Centrin3 expressing cells (Fig. 7), the localisation of GFP-Centrin 3 is modified: the intensity of the Centrin3 spots is weaker on new basal bodies assembled after the onset of gene inactivation (Fig. 7A-D). Therefore, ICL1e is required for Centrin3 localisation.

To analyse further this decrease in GFP-Centrin3 fluorescence, a STED investigation was carried out to localise the GFP-Centrin3 spots more precisely. It appears that GFP-Centrin3 has a triple localisation: one spot sits at the tip of each basal body (Fig. 7B inset) and two spots at its base (Fig. 7E,G). These labels fit well previous ultrastructural observations, where the double proximal (Ruiz et al., 2005) and the tip localisations (Aubusson-Fleury et al., 2012) were observed. Upon ICL1e knockdown, at an early stage, the sole right basal spot (Fig. 7F,H) disappears, while the tip and basal left spots will disappear during the next duplications. The fact that all Centrin3 spots do not disappear concomitantly suggests that there are several pathways for Centrin3 recruitment at the basal body. The ICL1e centrin is required for the assembly of the right Centrin3 spot, while it is not for the left one, which identifies the site of assembly of the anterior left filament (ALF), a Centrin3-dependent transient appendage which guides the positioning of the daughter basal body (Jerka-Dziadosz et al., 2012).

Centrin2 and Centrin3 are sequentially required for the maturation and positioning of the basal bodies (Ruiz et al., 2005): the present observations add one actor along the functional sequence, Cen2-ICL1e-Cen3. This interdependence between the three basal body centrins suggests the existence of physical connections, likely ensured by centrin-binding proteins of the Sfi1 type, homologous to the Sfr13 described in *T. thermophila* and shown to affect basal body duplication and anchoring (Stemm-Wolf et al., 2013; Heydeck, et al., 2016).

In view of this essential function of the ICL1e centrins in basal body anchoring and organisation of the ciliary rows and of their intricate role with the other ubiquitous centrins, it seemed of interest to ascertain their phylogenetic position in relation to the other centrin families.

### Centrin diversification during eukaryotic evolution

**Fig. 8.**
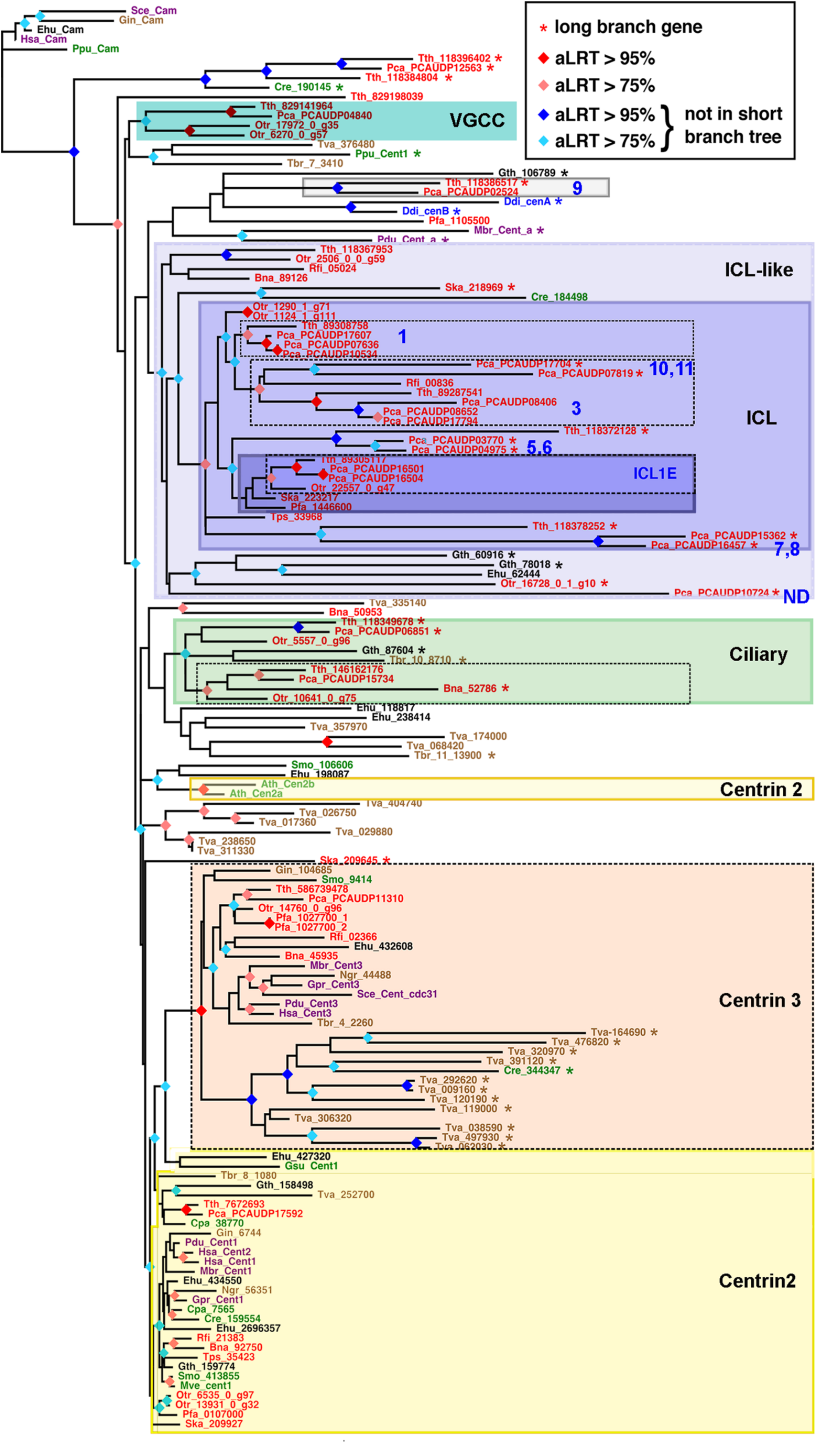
**Maximum-likelihood tree of centrins.** The colours of the gene labels indicate the lineage of origin (red, SAR; brown, Excavates; green, Archaeplastids; blue, Amoebozoans; purple, Opisthokonts). The ICL subfamilies of *Paramecium* are noted in blue numbers. ND, not determined. Species abbreviations: Ath, *Arabidopsis thaliana*; Bna, *Bigelowiella natans*; Cpa, *Cyanophora paradoxa*; Cre, *Chlamydomonas reinhardtii*; Ddi, *Dictyostelium discoideum*; Ehu, *Emiliana huxleyi*; Fi, *Retyculomyxa filosa*; Gin, *Giardia intestinalis*; Gpr, *Gonapodya prolifera*; Gsu, *Garderia sulphuraria*; Gth, *Guillardia theta*; Hsa, *Homo sapiens*; Mbr, *Monosiga brevicolis*; Mve, *Marsilea vestita*; Ngr, *Naegleria gruberi*; Otr, *Oxytricha trifallax*; Pca, *Paramecium caudatum*; Pdu, *Platynereis dumerii*; Pfa, *Plasmodium falciparum 3D7*; Ppu, *Porphyridium purpureum*; Sce, *Saccharomyces cerevisiae*; Ska, *Symbiodinium kawagutii*; Smo, *Selaginella moellendorffii*; Tbr, *Trypanosoma brucei*; Tps, *Thalassiosira pseudonana*; Tth, *Tetrahymena thermophila*; Tva, *Trichomonas vaginalis*. Stippled black boxes indicate well supported phylogenetic groups (red and orange nodes): Centrin3, Ciliate ciliary centrins, and ICL centrins corresponding to the *P. tetraurelia* ICL1, ICL3/10/11 and ICL1e centrins. Potential extension of these well supported groups with reduced tree support (blue nodes) are indicated in colours (blue for ICL/ICL-like and green for ciliary centrins). The group of Centrin2 sequences is only weakly supported and some sequences jump out of this group. This is for example the case in *Arabidopsis* in which the two centrin genes, not considered as Centrin2 by some authors (Hodges et al., 2011), nevertheless display some of its functional properties (Molinier et al., 2004; Nishi et al., 2013).

The present analysis (Fig. 8) updates previous phylogenetic analyses (Ruiz et al., 2005; Azimzadeh and Bornens, 2007; Shi et al., 2008; Gogendeau et al., 2008) and integrates genomes from all major eukaryotic groups, as recently described (Adl et al., 2012). In addition to *Chlamydomonas* *rheinardii* and *Saccharomyces* *cerevisiae* in which the founding members of the Centrin2/vfl2 and Centrin3/cdc31families were discovered, we exclusively screened completely sequenced genomes (Table S1). As expected, only one centrin gene was detected in *S. cerevisiae*, but contrary to the prevalent idea of a single centrin in *C. rheinardtii*, we found two additional centrin genes. In other species, the number of centrin genes identified is highly variable, up to 22 in the Ciliate *Paramecium* *caudatum* and even 24 in the Parabasalia *Trichomonas vaginalis*. This is a first indication that the story of centrins in the course of eukaryote diversification has been complex, with multiple duplication events in some branches and gene losses in others.

Strikingly, the resulting maximum-likelihood tree (Fig. S4-1) shows branches leading to individual genes of extremely variable length, not only across lineages but also in the same species. This is an indication that evolutionary constraints on primary structures have been highly variable between different gene types, maybe due to the number and flexibility of the molecular contacts that centrins make in the cell.

To ascertain the robustness of groupings, we compared the complete taxa sampling tree (Fig. S4-1) with a smaller tree, comprising only those genes that have evolved more slowly (see Materials and Methods section). The tree (Fig. 8) supports unequivocally only a limited number of groupings containing genes from several lineages, thus likely to represent ancestral eukaryotic genes. The monophyletic group comprising all Centrin3/cdc31 genes is particularly obvious, one of the two additional *Chlamydomonas* centrin genes unambiguously clustered in this group, confirming our previous analysis (Ruiz et al., 2005). A group containing short branch Centrin2/VFL2 sequences from a number of lineages is only weakly supported and some genes, interpreted as orthologous to Centrin2 for functional reasons, do not cluster with them. This may be interpreted as follows: an ancestral Centrin2 gene (with a high constraint on sequence evolution) is a certainty, owing to the diversity of lineages found in the weakly supported group. However, many other derived centrins (the long-branch genes) may have originated by duplication from this ancestral gene, thus making the functionally conserved Centrin2 a paraphyletic stem group. The derived centrins do not cluster with Centrin2 in this tree because of their exuberantly high evolutionary speed. They instead branch deeper or group together due to long-branch attraction.

In favour of this scenario no other well-supported monophyletic group, with centrins coming from multiple lineages, appears in this tree. All other groupings mostly comprise long-branch genes. This is for example the case for one additional node which regroups several ciliate genes, including *Paramecium* ciliary centrin genes, and a long branched cercozoan gene. In some cases, such as the *Trichomonas* centrins, all sequences of the node come from the same species, suggesting multiple gene duplications as already demonstrated (Carlton et al., 2007) and a rapid divergence of a paralogous gene sub-family. In other cases, when several eukaryotic lineages are represented, the statistical support of the node is low and the grouping dubious.

### Origin of the ICL /ICL-like centrin genes

Does our tree suggest an evolutionary origin of the ICL genes? Most, but not all, ICL sequences appear as long-branch genes. This indicates that they evolved rapidly with relaxed constraints as compared to the highly constrained Centrin2 and Centrin3 proteins. Retracing their evolutionary history is therefore difficult. The ICL sequences do not form a monophyletic group and may have originated from independent events of gene duplications from an ancestral centrin.

However, an important grouping, statistically supported and robust to resampling, emerges (ICL in Fig. 8). This branch groups not only many ciliate genes but also some genes from other species of the Stramenopile/Alveolates/Rhizaria (SAR) lineage (Adl et al., 2012). By contrast, a lone green algal sequence (*Chlamydomonas* 184498) jumps out of this group in the short branch tree, casting doubt on its affinities with ICL genes; but, even if no other plant sequence matches this additional branch, a sequence highly similar to this *Chlamydomonas* sequence is found in *Volvox* (Prochnik et al., 2010), another Chlorophyte able to divide in its flagellated stage, suggesting that this property could put a constraint to retain ICL-related genes.

This ICL grouping may in fact be even larger as a broader group emerges. In addition to the *Chlamydomonas* and Dynophyta sequences, this larger ICL-like group contains genes coming from another SAR lineage, as well as from potentially SAR-related lineages, Cryptophytes and Haptophytes. Next to this group is another branch with again a formally identified ICL gene from *Paramecium* (ICL9), suggesting that this branch could also correspond to ICL/ICL-like genes. Although the group does not show a strong statistical significance, it is striking to find in this branch one sequence of another Ciliata (*Tetrahymena*). These groupings indicate that at least one ICL gene may have appeared by duplication of an ancestral centrin gene (either Centrin2 or Centrin3) in an ancestor of the SAR lineage or an even larger lineage. Finally, the occurrence of sequences from organisms of other lineages (Cryptophyta, Dictyostelia, Apicomplexa), and more unexpectedly from one Metazoon (*Platynereis*) and the Choanozoon (*Monosiga*), show that some divergent centrins also exist in these organisms.

Within the ICL-like groups, few groupings emerge robustly. One is the ICL1e subfamily of ciliates. Interestingly, these particular genes have relatively short branches, indicating strong evolutionary constraints on their primary structures. Two similar short-branch genes from the dinoflagellate *Symbiodinium* and from the apicomplexan *Plasmodium* cluster with these ciliate centrins, although with a low significance. This suggests that a distinct ICL1e gene may have already existed in the ancestors of Alveolates. Besides ICL1e, two other well supported groupings are recognised within the ICL, one comprising only ciliate sequences, the other one comprising ciliate sequences and one foraminiferan ICL sequence, suggesting further that some diversification by gene duplication of the ICL had already taken place in the ancestor of the SAR lineage.

## DISCUSSION

In *P. tetraurelia*, the functional and molecular dissection of the ICL had led to the characterisation of a large number of centrins, phylogenetically distinct from the major Centrin2 and Centrin3 families (Gogendeau et al., 2008). We demonstrate here that the ICL1e centrins, one subfamily of the ICL centrins, contribute to the correct insertion and ordering of the basal bodies at the cell surface through an interaction with the ubiquitous centrins, Centrin2/Vfl2 and Centrin3/cdc31. In addition, these proteins, which form a complex associated with the basal bodies, constitute the organising centre of the ICL.

In view of this essential role in basal body duplication and in maintenance of ciliary planar polarity, we revisited their evolutionary status. The existence of three ancient families previously identified (Ruiz et al., 2005; Azimzadeh and Bornens, 2007) is confirmed with a broader sampling: the centrins 1, 2 and 3 are unambiguously ancestral eukaryotic proteins. ICL centrins have a more recent origin but are still very ancient, as they are shared by the whole SAR lineage and even possibly a broader clade of eukaryotes. Among ICL centrins, the ICL1e sub-family emerges as particularly ancient, conserved and constrained.

### The essential functions of the ICL1e centrins

We show here that a basal body-associated complex, the ICL1e complex duplicates along with the basal bodies and that the sequential recruitment of the Centrin2, ICL1e and Centrin3 is required for correct duplication and positioning of the new basal body. The effects of ICL1e deficiency resemble those resulting of Centrin3 deficiency (Ruiz et al., 2005), as in both cases, the unanchored basal bodies appear well developed.

The genus *Paramecium* has undergone several whole genome duplications (WGD) in the course of evolution (Aury et al., 2006), and the comparison between different species allows us to trace the history of the ICL1e subfamily. In *P. caudatum*, which diverged from *P. tetraurelia* before the last two WGDs, only two ICL1e centrins were identified. Although not functionally studied, they can be assumed to have the same role as in *P. tetraurelia*. One ICL1e gene was also detected in two other Ciliates, *Tetrahymena* and *Oxytricha*. In *Tetrahymena*, this gene corresponds to the *Ttcen4* gene encoding a centrin which also localises at basal bodies (Stemm-Wolf et al., 2005). No functional analysis is available in this genus, but the similar localisation of this ICL1e suggests the same function in the two Ciliates. No functional data are available for the ICL1e in *Oxytricha*, but data obtained in another related species (Lemullois et al., 2004) suggest similar functions for centrins in this lineage.

Surprisingly, orthologs of ICL1e are also present in the two other groups of Alveolata, but not in any other group. The common cytological particularity of Alveolata is the presence of alveoles, which form a layer beneath the plasma membrane (Stelly et al., 1991). Basal bodies and extrusomes are anchored to the plasma membrane between two adjacent alveoles. Thus, to anchor, the new basal body has to be positioned very precisely not only with respect to its parent, but also to the superficial surrounding architecture. Our observations strongly suggest that, in addition to Centrin3 required to build the ALF that guides the new basal body during its assembly (Jerka-Dziadosz et al., 2012), ICL1e is necessary to recruit Centrin3 in the proximity of the striated fibre where the pro-basal body is assembled during duplication (Iftode and Fleury-Aubusson, 2003). One hypothesis is that this additional system may generate an additional fine tuning during basal body positioning.

### Centrin diversification and contractility

It is quite surprising that a large part of the centrin diversity in *P. tetraurelia* is represented by ICL centrins, required for the assembly of the infraciliary lattice. In *P. tetraurelia*, this diversity represents up to 38/50 genes, and the expression of all is required for the assembly of this network. The fact that this network seems to be dispensable for cell survival, at least under laboratory culture conditions, raises the question of how and why such a diversity has been retained in the course of evolution.

In *P. caudatum*, the ICL centrins represent 16/21 centrin genes, corresponding to the 10 subfamilies defined in *P. tetraurelia*. In our broad centrin phylogeny, two genes, each representing one subfamily (ICL8 and ICL9), exhibit long branches suggesting a rapid evolution. In addition, four subfamilies (ICL3a, ICL3b, ICL10 and ICL11) appear closely related, suggesting a more recent divergence. Interestingly, in *Tetrahymena thermophila*, which displays a general organisation similar to that of *Paramecium* but a centrin network reduced to an apical band (Jerka-Dziadosz, 1981; Jerka-Dziadosz et al., 1995), five ICL genes have been unambiguously detected corresponding to the five *Paramecium* sub-families. In the other distant species devoid of ICL, *Oxytricha trifallax*, the only unambiguous ICL gene, belongs to the ICL1e subfamily. These results suggest that the centrin diversification observed in *Paramecium* could be related to the complexity of the infraciliary lattice.

Contractile networks of centrin in Ciliates are one of the three main components of the superficial cytoskeleton (Fleury et al., 1992). It can thus be assumed that this centrin-based contractility is ancestral and could ensure a widespread function in Ciliates, such as a primary escape mechanism, by contraction especially evident in some species (Misra et al., 2010). The multiplicity of the centrins mediating contraction of the cell cortex is to be compared to that of the protagonists of actin networks in metazoa (Pollard, 2016), suggesting that contractile systems need multiple actors to be effective. In some species, the centrin network forms a thick layer in which basal bodies (Viguès et al., 1984), as well as pro-basal bodies (Furness and Butler, 1985), are inserted. Although in such species, the function of this network has not yet been ascertained, its development, organisation and behaviour suggest that it would be required not only for contractility, but also for the transmission of the basal body pattern during the cell cycle. The fact that such a complexity of centrins is retained in *Paramecium* for a network dispensable for main cell functions could correspond to remnants of its evolutionary history. Alternatively, the network could be required under specific environmental conditions.

### Which centrins for which organisms?

Based upon an extensive sampling of eukaryotic lineages, the present phylogenetic analysis confirms the diversity of centrins beyond the most conserved families, Centrin2/Vfl2 and Centrin3/cdc31, currently recognised in eukaryotes (Azimzadeh and Bornens, 2007). It leads to the following general observations: (1) Only two centrin genes, Centrin2 and Centrin3, were undoubtedly already present in the last common ancestor as suggested by previous phylogenies (Azimzadeh and Bornens, 2007). Our phylogeny confirms that Centrin2, considered as a core component of the centriole (Hodges et al., 2010), is maintained in organisms with basal bodies and lost or present but highly divergent in the other ones. In the few groups which have lost Centrin3, at least another divergent centrin is found in addition to the canonical Centrin2, suggesting that representatives of several centrin families are necessary to ensure the continuity of basal bodies (Fig. S4-2). (2) Additional centrins have appeared in other lineages. In some lineages, one or few additional centrins are detected but in two lineages (Ciliata and Parabasalia), whole new families comprising many genes have evolved. The tree presented here suggests that either Centrin2 or Centrin3 could have duplicated.

Among these divergent centrins, the ICL proteins are particularly remarkable. A large proportion of ciliate ICL centrins emerges as a single group comprising molecules found in the SAR lineage (or even from a larger SAR/Chromalveolate grouping). The tree thus suggests that at least one ICL-like molecule appeared early in eukaryotic evolution. The particular conservation and functional specialisation of one particular sub-type, ICL1e, suggests a scenario for the functional diversification of these ICL-like molecules. We show that ICL1e centrins play a crucial role in basal body anchoring in ciliates. The occurrence of closely related ICL1e-like molecules in other SAR groups indicates that ICL1e evolved by gene duplication in the SAR ancestor and could have retained its ancestral function in basal body maintenance. Later on, additional duplications of this ancestral ICL type took place but the new paralogs diverged further, evolved more rapidly and were recruited for building evolutionary novelties, like centrin networks of ciliates. Because of its conserved function at the basal body, the ICL1e centrin has remained more constrained than the other paralogous ICL proteins. This scenario remains tentative as the function of potential ICL1e orthologs in groups other than ciliates remains to be explored.

Finally, besides the SAR group, other organisms display divergent centrins which could have other functions. In *Trypanosama brucei*, the phylogenetically divergent centrin detected here has been shown to interact with one basal body centrin (Wang et al., 2012), like ICL1e in *Paramecium*, and to coordinate cell and nuclear division (Shi et al., 2008). In *Chlamydomonas*, where we detected one ICL-divergent gene in addition to Centrin2 and Centrin3, centrin is also an important component of the basal body-nucleus connector (Geimer and Melkonian, 2005; Dutcher and O'Toole, 2016). Finally, and unexpectedly, one divergent centrin is also found in two Holozoa, *Monosiga* and *Platynereis*. It is not surprising that *Monosiga* displays one additional centrin: in most groups where cells are able to divide in their ciliated/flagellated stage, at least three different centrins are detected. The presence of a divergent centrin in a metazoon, *Platynereis* is more surprising. Indeed, the ability of ciliated cells to divide has not yet been detected in this lineage, where multiciliated cells correspond to a terminal differentiation (Brooks and Wallingford, 2014). To what extent the presence of a divergent centrin in *Platynereis* could be related to the existence of specific pathways of ciliation of its larval stage remains to be explored.

## MATERIAL AND METHODS

### Strains and culture procedures

Three strains were used: the wild-type reference strain d4-2 of *Paramecium tetraurelia*, the mutant nd7-1 (Skouri and Cohen, 1997) and the strain Δ-CenBP1/nd7-1 (Sehring et al., 2009). Cells were grown at 27°C in a wheat grass infusion (BHB, L'arbre de vie, Luçay Le Male, France) bacterised with *Klebsiella pneumoniae* and supplemented with 0.4 µg/ml β-sitosterol (Sonneborn, 1970; Beisson et al., 2010).

### Gene cloning

Genes coding for centrin were amplified by PCR (using the oligonucleotides listed in Fig. S3-1) and introduced in appropriate restriction sites of the silencing plasmid or *Paramecium* expression vectors. Accession numbers: *PtICL1e*: GSPATG00007681001; *PtICL1g*: GSPATG00021508001; *PtCen8*: GSPATG00008885001; *PtCen10*: GSPATG00003386001; *PtCen12*: GSPATG00000011001; *PtCen15*: GSPATG00001075001; *PtCen18*: GSPATG00038985001. Amplifications were performed with high fidelity Phusion DNA polymerase (Thermo Scientific) according to the manufacture's protocol.

### GFP constructs

GFP coding sequence was 5′-inserted in each centrin gene and cloned in the KpnI site of the expression vector pPXV-GFP (Gogendeau et al., 2005). GFP-fusion constructs were placed under the control of the *Paramecium* calmodulin regulatory sequences with 4-6 nucleotides coding for glycine amino acid introduced between the GFP coding sequence and the centrin genes.

### Obtaining transformed cell lines

ND7 cells were transformed by microinjection (Beisson et al., 2010). For each gene, at least 10 transformed clones were analysed.

### RNAI by feeding

T7Pol-driven dsRNA were produced from at least 75% of the genomic sequence (Fig. S3-2) from each centrin gene amplified by PCR and cloned into the L4440 feeding vector (Timmons and Fire, 1998). Feeding experiments were performed as described (Beisson et al., 2010). Possible RNAi-off target effects were searched for each construct. As off-target sequences were detected using tools available at ParameciumDB (Arnaiz et al., 2007) (Fig. S3-2), controls of feeding efficiency and/or specificity were performed by analysis of GFP-fluorescence decrease after feeding of clones expressing the GFP-ICL1e centrin with constructs used for inactivation of ICL1e (Gogendeau et al., 2008), PtCen8/PtCen15, PtCen12/Cen10, and Cen18: significative GFP decrease was observed only after ICL1e inactivation (not shown), thus assessing the specificity of the RNAi. At least five independent experiments were carried out for each gene analysed.

### Immunostaining

Cells were immunostained as previously described (Beisson et al., 2010). The antibodies were used at the following dilutions: anti-ICL centrin 1A9 culture supernatant (Beisson et al., 2001) 1:200, monoclonal anti-tubulin antibody culture supernatant 1D5 (Wehland and Weber, 1987) 1:100, the polyclonal PolyE anti-tubulin serum (Janke and Bulinski, 2011) 1:500, anti-GFP antibody from Interchim (Montluçon, France) 1:1000 and secondary antibodies labelled with AF488 or AF568 (Invitrogen-Molecular Probes, Eugene, OR, USA), or Cy5 (Life Technologies) diluted 1:200-1:500. Each experiment was done on samples of 50-200 cells.

### Fluorescence microscopy

Cells were observed with an epifluorescence Zeiss Axioskop 2. Images were acquired with a CoolSnap camera coupled with Metavue. Confocal acquisitions were made with a Leica SP8 equipped a UV diode (line 405), and three diodes laser (lines 488, 552 and 635) for excitation and two PMT detectors. Images stacks were processed with Fiji and Photoshop.

STED imaging was performed using a Leica TCS SP8 STED 3X (Leica Microsystems CMS GmbH, Mannheim, Germany) equipped with a 100×1,4 Oil STED white objective, a WLL ranging from 470 to 670 nm for excitation and with three STED lasers at 592 nm, 660 nm and 775 nm. GFP and AF568 were excited at 488 nm and 561 nm and detection range were 500-550 nm and 575-625 nm respectively. STED was performed at 592 nm for GFP and 775 nm for AF568. Signals were recorded on Leica HyD Hybrid Detectors using time gated detection of 1.5-6.0 ns and 0.5-6.0 ns for GFP and AF568 respectively. A pixel size of 25 nm was used. For deconvolution, SVI Huygens was used.

### Electron microscopy

Cells were prepared as previously described for morphological observations (Aubusson-Fleury et al., 2012). Ultrathin sections were examined with a Jeol transmission electron microscope at 120 kVolt.

### Phylogenetic analysis

Screenings for centrin-like sequences was conducted on databanks of predicted peptides from whole genome sequencing (Table S1). In a few cases, this genome screen was complemented by an assembled transcriptome screen. Blast searches were conducted either online or with NgKlast v4.0 (Korilog, France). The sequence probe used for all blast searches was a concatenation of human centrin 1, 2 and 3. All hits above an E value threshold set at 1E-10 were reciprocally blasted against the human proteome and all those whose first human hit was a centrin, even with a low E value, were retained for further phylogenetic analyses. Five calmodulin-like sequences from different eukaryotic species were added as outgroups. A total of 149 sequences were thus retained. Sequences were aligned with the Muscle algorithm of Seaview (Gouy et al., 2010) and only the unambiguously aligned parts of the molecules were used for tree making. Alignments are available upon request to G.B. Tree making was performed with the Maximum Likelihood algorithm, as implemented in Seaview, using the LG model of amino acid substitution, no invariable sites, six categories of rate variations and aLRT scores for node robustness.

The multiplicity of the sequences, the relative shortness of the protein (only 158 aa positions were retained for tree making) and the large variations in evolutionary rates result in a tree in which many nodes, especially deep ones, have relatively low statistical value, as indicated by aLRT scores (Anisimova and Gascuel, 2006). To evaluate further node robustness, a selection of fast-evolving sequences was made. To this end, a table of Poisson distances between sequence pairs was calculated using the Distance Methods option of Seaview and the average distance of each centrin-like molecule to the calmodulin outgroup sequences (Table S2). We set arbitrarily a threshold at a value of 1.1 above which sequences were considered fast-evolving and prone to artefactual. This threshold resulted in the elimination of 43 sequences (29%) and another tree was built with the resulting set and the same options as above.
